# Supplementary material for: Learning curve and surgical outcome of robotic assisted colorectal surgery with ERAS program
Source: Sci Rep. 2022 Nov 29;12:20566. doi: 10.1038/s41598-022-24665-w (PMC9709162; doi:10.1038/s41598-022-24665-w)
Supplement: Supplementary file 1 — Supplementary Information 1. [file 41598_2022_24665_MOESM1_ESM.zip › LSC Raw data-Ñ[▒K/061/N_ñw╝╨░O▒Kñσ.pdf]

W55 / W55

臺中榮民總醫院
手術室護理記錄

[redacted]

基本資料

- 室別： 二醫手術室-36室，手術科別： CRS，手術類別： 常規，日期： 2020/01/10
- 進入時間： 1445 ； 麻醉開始時間： 1450 ； Time Out時間： 1540 ； 劃刀時間： 1542 ； 手術結束時間： 1930 送出時間： 1935 ；
- 麻醉方式： GE

  診斷： Malignant tumor of rectum,DENTATE LINE

  手術名稱： 1.Low anterior resection with colo-anal anastomosis2.Ileostomy3.4.

  術前預防性抗生素：
  - 抗生素名稱 ： Veterin 1GM IVD/由麻醉護理師給予
  - 時間： 1530
  - 上次簽章: 朱詠暄

  ，追加預防性抗生素：
- 手術醫師： 蔣鋒帆
- 麻醉醫師： VS 施涵薰
-
  - 刷手護士A ： 賴于帆，時間： 1445-1700，接替刷手一： 朱詠暄，時間： 1700-1730，接替刷手二： 王毓均，時間： 1730-1935
  - 流動護士A ： 朱詠暄，時間： 1445-1700，接替流動一： 林宜旻，時間： 1700-1800，接替流動二： 謝昀安，時間： 1730-1800
- 更多接替及流動護士： 接替流動三： 朱詠暄，時間： 1800-1930

皮膚狀況

A-V Shunt： 無

術前： 完整

記錄者： 上次簽章: 朱詠暄

術後： 完整

記錄者： 朱詠暄

手術準備

手術臥式 單一手術臥式

-
  - 手術臥式： 截石臥式
  - 使用附件： 溫毯 38 ℃，頭圈，腳架
  - 皮膚準備： 10%Aq Betadine，Hibiscrub，其它： 2%chlorhexidine
  - 電燒： 使用電燒: 電極片位置： 右大腿，記錄者： 上次簽章: 朱詠暄

止血帶T： 無

植入物

- 植入物名稱/號碼/數量/部位： surgiwrap(小)/壹/腹部

，記錄者： 朱詠暄

標本送檢

- 細菌培養 無
- 細胞學 無
- 冰凍切片 無
- 病理標本

  組織名稱一： rectum

  ，

  組織名稱二： anterior wall

  ，

  組織名稱三： IMA root
- 截肢 無

標本交付 標本： 參 袋 ，標本： 0 盒 ，標本： 0 瓶 ，細胞學： 0 管 ，Culture Tube： 0 管 ，被交付醫師： R 林陳彥，

- 記錄者： 朱詠暄
- 時間： 1935

傷口術後數料

傷口數料：

- 請於圖上標示部位：

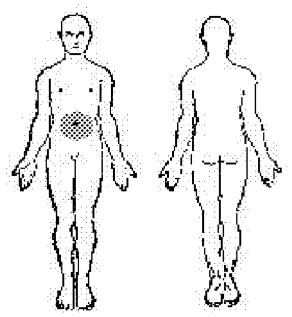

腹部參切口+肛門

- 傷口數料： mepore覆蓋
  肛門塞止血棉，散紗覆蓋布膠固定

導管

導管	天數	導管資訊	插入日期	插入時間	更換日期	拔除日期	拔除時間	拔除原因	預計拔除/更換	預計拔除/更換日期
Foley(1)	1	號碼:16 Fr.; 部位:膀胱; 註記:水球8CC;	2020/01/10	1520						
Sump NG Tube(1)	1	號碼:16 Fr.; 深度:60cm; 部位:胃;	2020/01/10	1525						
Penrose Drain(1)	1	號碼:12 mm.; 部位:骨盆腔; 註記:左腹;	2020/01/10	1800						

用物計數

- 手術中出血量： minimal c.c.
- 手術中尿量： 300 c.c.
-
  - 其它： 無

用物計數： 多切口同時縫合

- 1.器械： 正確，2.縫針： 正確，3.刀片： 正確，4.紗布： 正確，5.腹部墊： 未使用，6.紗球： 未使用，7.PEANUTS ： 未使用，8.紗條： 未使用，9.絲帶： 正確，10.針頭： 未使用，11.Vessel loop： 未使用，12.Boots： 未使用，13.Jelco： 未使用，14.棉條： 未使用，
  - 第一次：
    - 刷手護士 ： 王毓均
    - 流動護士： 朱詠暄
    - 時間： 1830

  ,
  - 第二次：
    - 刷手護士 ： 王毓均
    - 流動護士： 朱詠暄
    - 時間： 1850

  ,
  - 第三次：
    - 刷手護士 ： 王毓均
    - 流動護士： 朱詠暄
    - 時間： 1910

備註

備註： NIL

- 記錄者： 朱詠暄 上次簽章: 朱詠暄
- 時間： 1935

填寫護理人員 朱詠暄

正式摘要

W55 / W55

doctorRank:B
